# Recent Developments in Topical Wound Therapy: Impact of Antimicrobiological Changes and Rebalancing the Wound Milieu

**DOI:** 10.1155/2014/819525

**Published:** 2014-04-15

**Authors:** Cornelia Erfurt-Berge, Regina Renner

**Affiliations:** Hautklinik Erlangen, Universitätsklinikum Erlangen, Ulmenweg 18, 91054 Erlangen, Germany

## Abstract

Wound therapy improves every year by developing new wound treatment options or by advancing already existing wound materials, for example, adding self-releasing analgesic drugs or growth factors to wound dressings, or by binding and inactivating excessive proteases. Also new dressing materials based on silk fibers and enhanced methods to reduce bacterial burden, for example, cold argon plasma, might help to fasten wound healing.

## 1. Introduction

Optimal modern wound dressings should assure a moisture wound bed, help drainage, remove debris of the wound surface, provide optimal thermal stability, might be removed without trauma of the wound bed and wound edge, and be antiallergenic and without immunogenicity. But wound dressings have experienced continuous and significant changes over the years which emanate from a more detailed understanding of wound healing and improved technological, clinical, and scientific research in the field of wound healing. Now, wound dressings are getting more and more functionalized to a targeted therapy by including different pharmaceutical compounds (e.g., antiseptics, analgetics, or growth factors). Those interactive additives might help optimize the healing process.

Here, we want to present an overview about some of those new developments in topical wound therapy (see also Table 1).

## 2. Debridement

Debridement is a basic necessity to reduce fibrin and bacterial burden on the wound surface and is therefore a kind of wound bed preparation to (re)induce wound healing. There are different mechanical debridements available (surgical/sharp debridement, ultrasound, and hydrosurgery), as well as autolytic debridements via hydrogels, enzymatic dressings, or honey, and a biosurgical debridement via larval debridement.

Autolytic debridements are normally well tolerated because they cause no or only very little pain, but it takes much more time in contrast to mechanical debridement to reduce slough and fibrinous burden on the wound. Honey might be a little bit more painful in this context due to its osmotic action. On the other hand, honey might be additional antimicrobial effective by this osmotic dehydration, a low pH-value of 3.0–4.5, and the release of small amounts of hydrogen peroxide or methylglyoxal [1].

Normally, mechanical debridement is easy and fast but on the other hand not very selective and for the patient sometimes very painful procedure. A recently induced more gentle method to reduce mechanically slough and fibrinous burden from the wound bed is a monofilament fibre product whose wound-contact side is fleecy and if wetted it has to be wiped over the surface of the wound for 2–4 minutes [2]. Its advantage is that it is a quick method causing only little or no pain [3].

Larvae debridement therapy is very selective to necrotic tissue, in most patients also painless, and due to the larval digestive juices secreted by the larvae antimicrobial effective and pH-modulating [3].

## 3. Analgetics

Pain is a well-known problem in patients with chronic leg ulcers, especially for elderly patients where it may lead to reduced wound healing rates and reduced quality of life [4]. Causes for wound pain are the underlying pathology of the leg ulceration, the wound itself, wound treatment including dressing changes, or complications such as skin irritation around the ulcer. Therefore, reducing pain represents a major part of attentive wound care and pain therapists should be involved in the wound care team. While wound pain is mainly treated by systemically applied analgetics, the topical treatment with analgetic-releasing dressings seems not to be used in its full capacity so far. To overcome side effects of systemically applied analgetics, a combination of moisture-balancing foam dressings with the release of low-dose nonsteroidal anti-inflammatory drugs (NSAID), that is, ibuprofen, was developed representing a new therapeutic class of local pain-relieving dressings. Gottrup et al. [5] evaluated the outcome of patients treated with ibuprofen foam in a randomized controlled double-blind clinical investigation. Wound pain intensity in patients with venous ulcers was significantly reduced with the ibuprofen foam compared to the pain-reducing effect of moist wound therapy alone. In addition to an early onset of pain relief after application of the ibuprofen foam dressing, a reduction of pain during dressing change could be achieved. These data were affirmed by another randomized controlled trial (RCT) in 2011 [6]. Topically applied NSAIDs also may be associated with local contact allergic reactions [7]. In the studies described above, no additional severe adverse reactions were observed during application, whereas adverse effects of systemic treatment of pain with NSAID include gastrointestinal bleeding or renal failure. Taken together, no significant recommendation for these dressings can be given as long as only a small number of RCTs exists and further investigations about side effects or systemic uptake are missing. But ibuprofen-releasing wound dressings can be seen as an individual supplement within the possibilities of analgetic therapy.

Beyond the possibility to add analgetics to wound dressings, the structure of the dressing itself can be used to reduce wound pain. Examples for this approach are silicone dressings which can be used to prevent dressing-related trauma or skin stripping while dressing change [8]. Dressings with hydro-balanced biocellulose were also shown to minimize wound pain significantly [9]. For instant, interventions, for example, sharp debridement, topical formulations with local anaesthetics like lidocaine-prilocaine cream, known as Eutectic Mixture of Local Anaesthetics (EMLA), represent an established pretreatment procedure and showed significant reduction in pain in several trials [10]. Another promising therapeutic alternative is a topical morphine gel which provides a moist wound therapy and reduced wound pain in 90% of treated patients significantly [11]. It can be used in preparing interventions or as a permanent pain-reducing wound treatment.

## 4. Growth Factors 

Growth factors (GF) have been shown to promote wound healing in acute wounds by stimulating the growth of keratinocytes. Since the process of wound healing depends on the mitosis and migration of keratinocytes, reduction of GF represents a key factor for chronification of wounds. Application of GF implemented into topical wound dressings represents one possible attempt to face this problem. The in vivo use of a bilayer wound dressing containing epidermal growth factor- (EGF-) loaded microspheres decreased wound areas in a rabbit model in a dose-dependent manner much faster than wound dressings without EGF [12]. Interesting results were also attained by a sodium carboxymethylcellulose-based topical gel, containing the active ingredient becaplermin, a recombinant human platelet-derived growth factor. Granulation tissue could be increased by stimulation of fibroblast proliferation and collagen production and healing time of diabetic ulcers was reduced by 32%, nearly 6 weeks faster than placebo gel [13]. In opposition to this efficacy, adverse side effects occurred with rise of mortality in cancer patients. As a consequence, distribution was shut down in Europe in 2011. Beyond this remarkable case of adverse events in the use of GF, the application of purified GF via a wound dressing raises technical difficulties with low bioavailability and high manufacturing costs, and the fact that wound healing requires a complex mixture of growth factors. But there are still developments to improve bioavailability of single or multiple growth factors in wound dressings, for example, by incorporating them into biomaterial based dressings like silk fibers or with release in a time-dependent manner.

## 5. Biological or Synthetical Fibers Used for Wound Dressings

Artificial engineered scaffolds for wound healing are mimicking structure and functions to native extracellular matrix. These scaffolds can be created by, for example, bacteria producing cellulose (the so-called biocellulose, e.g., by Acinetobacter Xylosis), by cultured transgenic silkworms or by several electrospinning techniques. The last one is an efficient technique to produce a variety of polymeric nanofibers. Electrospun scaffolds for wound healing provide high surface area to volume ratio that promotes cell-matrix interaction at the nanoscale [11, 14, 15]. Electrospun collagen is the most biomimetic nanofibrous scaffold due to its structure and origin closely similar to the native extracellular matrix of the skin, but also its alternative, gelatine, and other natural polymers including silk fibroin, chitin/chitosan, fibrinogen, and different blends have been electrospun as wound dressing or cellular matrix and have been demonstrated to be efficient to cell attachment, growth, and cell infiltration in in vitro culture [16]. Artificial created scaffolds also can be combined and added with different components like antibiotics or antibacterial drugs, anaesthetics like lidocaine [17], or Ag particles that are spun together with the synthetic nanofibers [18]. These combined scaffolds work as drug delivery systems and are releasing a controlled rate of the drugs and therefore might have great potential in different applications in wound healing. In the following, we highlight as an example silk based wound dressings.

## 6. Silk Based Wound Dressings

Wound dressings can be composed of synthetic or biologic materials. One of those with biologic materials is wound dressings made of silk. Silkworm silk fibers are composed of two fibrous protein microfilaments (fibroins) embedded in a glue-like glycoprotein coating (sericin) [19] and can be isolated from the silkworm* Bombyx mori* or* Antheraea mylitta*. The sericins ensure the cohesion of the cocoon by sticking the twin filaments together and constitute 25–30% of the weight of the fiber [20]. Finally the fiber is coated with a variety of other proteins that should protect the cocoon against microbes or other predators [21].

Silk represents a new family of advanced biomaterials as a basis for wound dressings [22]. It is getting more and more attention due to its good biocompatibility, slow degradability, hemocompatibility, and high mechanical strength [23], although silk sericin might be immunogenic for patients [24, 25].

Kanokpanont et al. [24] evaluated a novel silk fibroin-based bilayered wound dressing versus a commercial wound dressing. So, in this new wound dressing, a wax-coated silk fibroin woven fabric functioned as a nonadhesive layer, while the sponge made of sericin and glutaraldehyde-cross-linked silk fibroin/gelatin was used as a bioactive layer.

The wax-coated silk fibroin fabrics showed improved mechanical properties less adhesive characteristics than the commercial wound dressing. The sericin-silk fibroin/gelatin spongy bioactive layers showed homogeneous porous structure and controllable biodegradation depending on the degree of cross-linking. Furthermore, they proved that fibroin can improve wound healing by supporting of attachment of epidermal cells and fibroblasts spreading and proliferation [26, 27]. The wounds treated with the bilayered wound dressings showed more reduction of wound area and improved epithelization as well as collagen formation in full-thickness wounds.

There exist transgenic silkworms (*Bombyx mori*) that can combine their silk thread with human growth factors, for example, with epidermal growth factor (EGF), fibroblast growth factor (FGF), keratinocyte growth factor (KGF), platelet derived growth factor (PDGF), or vascular endothelial growth factor (VEGF) [28]. It has been shown that there was no cytotoxic effect of the silks and growth factors improved the proliferation of mesenchymal stem cells. EGF and VEGF additional supported angiogenesis of wound healing [29].

Also Schneider et al. [30] described the use of silk mats made of porous, structured electrospun nanoscaled silk fibers containing epidermal growth factor (EGF). EGF has high affinity receptors expressed in both fibroblasts and keratinocytes and has been shown to accelerate wound healing in vivo by stimulation of proliferation and migration of keratinocytes [31–33].

They demonstrated that EGF is incorporated into the silk mats and is slowly released in a time-dependent manner (25% EGF release in 170 h) initiated by a burst effect on keratinocytes on the wound edge. This can be explained by the fact that the protein is not only inside the fiber but also at the surface. The first increase of EGF concentration is due to the ones on the surface of the silk fibers, and the second part of the release profile shows a more smooth increase, while EGF is released due to diffusion of the EGF molecules from inside the silk fibers to the fiber surface.

So they could demonstrate in vivo with their human three-dimensional model that the biofunctionalized silk mats aid the healing by increasing the time of wound closure by the epidermal edge by 90%. Structure of the mats was preserved during the healing period.

Gil et al. [34] compared three different material designs and two different drug incorporation techniques for silk protein-biomaterial wound dressings with (a) epidermal growth factor (EGF) and (b) silver sulfadiazine in a cutaneous excisional mouse wound model. Material formats included silk films, lamellar porous silk films, and electrospun silk nanofibers; each studied with the silk matrix alone and coated/loaded with EGF or silver sulfadiazine. They compared those silk dressings with synthetic air-permeable Tegaderm Tape (3M) as well as Tegaderm Hydrocolloid Dressing (3 M). All silk biomaterials were effective for wound healing, but the incorporated silk fibres with EGF/silver sulfadiazine provided the most rapid wound healing responses. Also all functionalized silk biomaterial wound dressings increased wound healing rate, including reepithelialization, dermis proliferation, collagen synthesis, and reduced scar formation compared to the synthetic modern wound dressings.

## 7. Autologous Platelet-Rich Fibrin 

One alternative method to transfer endogenous growth factors to the wound area is the production of autologous platelet-rich fibrin (PRF) that is prepared from a simple blood sample of the patient and is therefore a really low burden to the patient. PRF contains fibrin and high concentrations of different growth factors. Using autologous material decreases the risk of adverse events like allergenic reactions and delivers a cocktail of different growth factors in comparison with topically applied single heterologous growth factors. Large systematic reviews of this therapy exist, but while one meta-analysis in which studies about acute wounds were also included found a sufficient efficacy to stimulate healing even in long-lasting wounds [35] and diabetic foot ulcers [36], another Cochrane Review analysing nine RCTs about chronic wounds could not show evidence for any superiority of PRF in comparison to standard wound care [37]. In conclusion, more well-designed studies are needed and PRF so far only represents a supplemental treatment option to standard procedures like debridement or an alternative to other wound healing promoting materials as described below.

## 8. Protease Inhibitors

While the deposition of GF on the wound area from external sources like wound dressings or via PRF could so far not sustain its position within standard wound treatments, the focus extended to the observation that chronic wounds have raised levels of protease activity and any GF that are applied to the wound are often subjected to proteolytic degradation which renders them inactive. In chronic wounds, matrix metalloproteinases (MMP) have been implicated by a number of studies as the major protease family responsible for the degradation of key factors critical to the ulcer's ability to heal [38]. While, in an acute wound the balance between proteolytic MMP and their natural antagonist, the tissue inhibitors of MMP (TIMP) promote the necessary amount of tissue degradation to clear the way for wound healing processes, wound fluid from chronic leg ulcers showed elevated levels of MMP [39, 40] and uncontrolled proteolytic tissue destruction which appears to be a pathogenic factor in nonhealing wounds [41, 42].

By binding and inactivating excessive proteases like MMP, endogenous growth factors are saved from degradation. For this reason, wound dressings with MMP inhibiting agents have recently been developed and are expected to improve the local treatment of these chronic wounds. Native bovine collagen type I was shown to bind proinflammatory cytokines and to decrease distinctively the concentration of MMP in wound fluids collected under treatment with a collagen-containing wound dressing [43]. A wound dressing consisting of collagen and oxidized regenerated cellulose showed a significant reduction of MMP and other proteases in chronic wound fluid from diabetic ulcers in an in vitro model [44] and was then compared to standard wound treatment in a randomized controlled trial where only slight significant benefit in ulcers with a duration of less than 6 months could be observed [45].

In another model, a nano-oligosaccharide factor (NOSF) was incorporated into a neutral Lipido-Colloid Technology (TLC) dressing and MMP inhibition by complex formation with TIMP could be demonstrated in vitro [46]. In direct comparison to the MMP-inhibiting collagen dressing described above the NOSF dressing showed significant stronger promotion of healing in venous leg ulcers [47]. In a double blind prospective RCT comparing NOSF dressing with a TLC dressing without NOSF wound area reduction in venous leg ulcers was achieved 2 times faster in the NOSF group [48].

In vitro experiments showed that polyacrylate superabsorbers were capable of binding MMPs directly and additionally reduced the enzymatic activity of MMPs by competitive ion binding [49].

Reducing protease activity in chronic wounds can only be one of several strategies in the treatment of stalling wounds. Proteases represent one of the best available biomarker to provide information about the status of a wound. Elevated protease levels in chronic wounds may not be characterized with other clinical criteria than presence of slough or fibrin. Therefore, diagnostic tests were generated, but additional research is recommended to evaluate the efficacy of such tests and the treatments and therapies applied when elevated protease levels are found [50, 51].

## 9. Antimicrobial Wound Therapy

Bacterial colonization of chronic wounds is common and a well-recognized factor contributing to impaired wound healing [52, 53]. To reduce bacterial load but to avoid the development of antibiotic resistance, the use of antiseptics or physical techniques is necessary [1].

### 9.1. Antiseptics

Antiseptics are used extensively to reduce bacterial load on the wound surface. Limitations for some antiseptics might be their cytotoxic and their allergenic potential. The most frequently used antiseptics without significant cytotoxic or allergenic potential are polyhexanide, octenidine, or silver-containing wound dressings. PVP iodine is used very often, too, because it is very cheap and effective but contains a relevant allergenic potential.

### 9.2. Silver Containing Wound Dressings

The broad antimicrobial effect of silver ions is based on complex formation with bacterial proteins leading to loss of function and structure of the bacterial membrane. Interaction with the enzymatic system and changes in the genetic information promote cell death. In vitro models showed a cytotoxic and wound healing delay in a mouse model. While the use of silver dressings has become very popular in standard wound care, a prospective randomized controlled trial (VULCAN) could not verify a significant effect on wound healing [54].

### 9.3. PHMB-Containing Wound Dressings

Similar inconsistent results were found in an RCT analysing treatment of chronic lower leg ulcers with a polyhexamethylene biguanide (PHMB) coated foam compared to standard foam dressings. While a significant reduction of wound pain and bacterial burden can be achieved, reduction of wound area could not be fastened [55]. Another study compared treatment of patients with critically colonised wounds with a PHMB-containing biocellulose wound dressing to treatment with a silver dressing and found that polyhexanide-containing dressings were more effective in reducing pain and bacterial burden. Data about wound area reduction were not collected in this study [56].

## 10. Cold Argon Plasma

In addition to the use of antimicrobial wound dressings, other physical strategies to remove bacterial burden are in use. A new and promising method in this physical field is cold atmospheric plasma [57]. In addition to solid, liquid, and gas phases, plasma is the fourth state of matter. The ionized plasma consists of a highly active mix of oxygen, nitrogen, and hydrogen radicals, ions, electrons, photons, and ultraviolet radiation. Plasma penetrates also hair follicles where disinfectants mostly fail to reach [58].

High-temperature plasmas are already in medical use for the sterilization of equipment, tissue destruction, cutting, and cauterizing. Cold atmospheric argon plasma has the same advantages as high-temperature plasmas but without heat production. An even plasma dose can be applied to different wound surfaces, and also larger wound areas (up to 6 cm diameter) can be treated within a single application [59].

Isbary et al. demonstrated that a 5-minute treatment in 38 wounds (291 treatments) [60] as well as a 2-minute treatment of 14 patients (70 treatments in total) [59] of the surface of chronic wounds resulted in a significant (34%, *P* < 10^−6^; 40%, *P* < 0.016) reduction of bacterial colonization in the plasma-treated wounds, regardless of the species of colonizing bacteria. No side-effects occurred and the treatment was well tolerated.

To proof if cold plasma treatment can also improve wound healing, forty patients with skin graft donor sites (acute wounds) on the upper leg were enrolled in a placebo controlled study [61]. The wound sites were divided into two equally sized areas that were randomly assigned to receive either plasma treatment or placebo (argon gas) for 2 minutes. Donor site healing was evaluated independently by two blinded dermatologists, who compared the wound areas with regard to reepithelialization, blood crusts, fibrin layers, and wound surroundings. Argon plasma treated wounds showed improved reepithelialization and fewer fibrin layers and blood crusts. The verum treated donor site (*n*  =  34) showed significantly improved healing compared with placebo-treated areas (day 1, *P*  =  0.25; day 2, *P*  =  0.011; day 3, *P* < 0.001; day 4, *P* < 0.001; day 5, *P*  =  0.004; day 6, *P*  =  0.008; day 7, *P* =  0.031). Wound infection did not occur in any of the patients, and no relevant side effects were observed [61].

But not only in acute wounds but also in patients with chronic wounds, the effect of cold plasma application was evaluated [62]. Chronic wounds of various etiologies in 70 patients were treated for 3–7 min with a subgroup analysis of chronic venous ulcers. Patient acted as own control. Wound dimensions before and after treatments were compared for plasma-treated and control wounds. All plasma-treated wounds with various etiologies showed a greater reduction in width and length than control wounds, but reduction rates were nonsignificant. In patients with venous ulcerations, a significantly greater reduction in width was measured in plasma-treated ulcers compared to controls, but not in ulcer length. So these results suggest that wound healing especially in chronic venous ulcerations might be improved by cold argon plasma treatment [62]. In summary, the advantages of cold argon plasma are its huge effectiveness to reduce bacterial colonization maybe because it also penetrates hair follicles, and without inducing resistances; and there are not known keratinocytes or fibroblasts toxic side effects, so it seems that there is no potential to inhibit wound healing by application. Last but not least, it is a fast and painless disinfecting method for the wound and the patient. Disadvantages might be the costs and the size of the device.

## 11. Wound Diagnostics

Sometimes it is very difficult to treat especially chronic wounds properly due to missing options for diagnostic measurements on different wound parameters. At the moment there are few options available in wound surface diagnostics, but in most cases therapeutic conclusions are still missing.

### 11.1. Measurement of Proteases Activity

A few years ago, a new measurement tool to detect elevated protease activity on the wound surface and in the wound fluid was developed: the so-called “woundchek” (Systagenix Wound Management LLC, Baden, Germany). Unfortunately at the moment, further therapeutic options to reduce or inhibit measured increased protease activity are still missing.

### 11.2. Measurement of pH-Value

Normally, healing wounds show a lower pH-value in contrast to a more basic pH-value of chronic nonhealing wounds [63]. Variations in pH-value can cause biochemical changes in the wound environment with a pH-range of 7.2–7.5 that seems optimal for tissue granulation [64]. Measurement of pH-Values is possible via litmus paper or by spot measurement via pH-electrodes.

In a recent study the pH levels of 4 different wound dressings (Manuka honey dressing, sodium carboxymethylcellulose hydrofiber dressing, polyhydrated ionogen-coated polymer mesh dressing, and a protease modulating collagen cellulose dressing) were compared regarding their effect on pH-value. All dressings were found to have a low pH (below pH 4). The lowest was the protease modulating collage cellulose dressing with a pH of 2.3. By this, the acidic nature of the analyzed wound dressings might influence the healing process in the wound [65].

Continuous in situ pH measurement on the wound surface is not possible at the moment due to a lack of suitable technologies. Normally, for pH monitoring, flat bottomed conventional glass pH probes are used. Recent innovations for wound pH sensing have developed a luminescent sensor spray with additional advantage of its applicability to uneven tissue surfaces due to its consistency. Images of the wound surface might result in pseudocolor pH maps of the area [66]. A new technology might be also printed composite electrodes for in situ wound pH monitoring [67].

### 11.3. Indicators for Bacterial Infection

All chronic wounds may be contaminated by microorganisms. But, in some cases, it is difficult to decide whether this wound is clinically infected or at least colonized with a critical bioburden of bacteria. Traditional criteria for wound infections are cellulitis and in most cases also abscess and development of pus.

In all other cases suggestive of critical or initial infection, it might be possible to seek secondary signs of infection. Some of them might be for venous leg ulcers increase in local skin temperature, increase or change in ulcer pain, newly formed ulcers within flamed margins of preexisting ulcers, wound bed extension within inflamed margins, or delayed healing despite appropriate compression therapy [68]. Those additional criteria were summarized in a position document of the EWMA via a Delphi approach. Some of them are similar to earlier postulated additional criteria [69].

## 12. Conclusion

Wound treatment has come a long way since its beginning and this is largely due to improved understanding of the physical characteristics of wound healing and the availability of smart wound dressing materials. Nowadays, wound dressings should not only serve as exudate managers or absorbents, and they should also interact on a cellular level to treat imbalances in wound milieu to improve wound healing.

Also new physical techniques and dressing materials like cold argon plasma or silk fibers found their way as innovative treatment options into wound therapy (see Table 1).

## Figures and Tables

**Table 1:** Overview of the presented wound therapies with classification of the level of innovation.

	Substance	State of the art (Germany)	Innovation level	Importance of the therapy	Hospital/out-patient use	Costs
Autolytic debridement	Wound dressing	Routine	Generic	+++	Both	+/++

Sharp mechanic debridement	Medical device	Routine	Break through	+++	Hospital	+

Mechanical debridement with Debrisoft	Medical device	Selected	Add on	+	Both	++

Growth factors	Drug	Selected	Break through	+	Hospital	+++

Electrospun scaffolds	Drug-device combination	Early development	Break through	+++	Hospital	+++

Silk based wound dressings	Drug-device combination	Early development	Generic	++	Hospital	+++

Platelet-rich fibrin	Drug	Selected	Add on	+	Hospital	+++

Protease inhibitor	Wound dressing	Selected	Break through	++	Both	++

Antiseptics in general	Drug/wound dressing	Routine	Break through	+++	Both	+/++

Silver	Wound dressing	Selected	Break through	++	Both	++

Cold argon plasma	Medical device	Selected	Break through	+++	Hospital	+++

Wound diagnostics: protease levels	Medical device	Selected	At the moment: add-on	++/+++	Hospital	++

Wound diagnostics: pH-values	Medical device	Selected	At the moment: add-on	++/+++	Hospital	+/++

